# *Verruconis gallopava*: an isavuconazonium foe

**DOI:** 10.1128/asmcr.00081-25

**Published:** 2025-07-31

**Authors:** Divisha Sharma, Scott R. Curry, Courtney E. Harris, Alexandra Mills, Rachel Burgoon, Drew W. Charles

**Affiliations:** 1 Division of Infectious Disease, Medical University of South Carolina, Charleston, South Carolina, USA; 2 Department of Pharmacy, Medical University of South Carolina, Charleston, South Carolina, USA; Rush University Medical Center, Chicago, Illinois, USA

**Keywords:** pneumonia, transplant, isavuconazole, *Verruconis gallopava*

## Abstract

**Background:**

*Verruconis gallopava* is a dematiaceous and environmental commensal that rarely causes infection in humans. Human infections are typically observed in immunocompromised individuals. Clinical presentations range from localized infectious (skin and lung) to disseminated disease with a predilection for the central nervous system. Currently, no standardized guidelines exist for the treatment of *V. gallopava*. Most mold-active triazoles, except isavuconazole, have demonstrated *in vitro* antifungal activity against this pathogen.

**Case Summary:**

We present a case of an immunocompromised male who underwent heart transplantation with subsequent syndrome of chronic pneumonia. Imaging suggested an atypical pulmonary process prompting empiric initiation of isavuconazonium. Despite treatment, the patient’s condition worsened, and fungal culture from sputum and bronchoalveolar lavage both grew *V. gallopava*. His symptoms persisted, and he was transitioned to liposomal amphotericin B and eventually oral posaconazole (POS) with clinical and radiographic resolution of his presenting syndrome.

**Conclusion:**

This case highlights the limited experience and clinical failure of isavuconazonium as empiric therapy for *V. gallopava*. Transitioning to POS led to clinical improvement, suggesting its potential as a treatment option. Further experience is needed to establish optimal therapeutic strategies for this rare but serious infection.

## INTRODUCTION

Dematiaceous (pigmented) fungi are commonly found as saprophytes in the environment, but rarely cause infection in humans, referred to as phaeohyphomycosis (1). *Verruconis gallopava* differs from other dematiaceous fungi, such as *Onchroconis*, primarily in its thermal preferences (2). *V. gallopava* is thermophilic, thriving in hot environments like thermal soils and hot springs, and can grow at temperatures up to 42°C (2). It is known for its neurotrophic potential, acting as an opportunistic pathogen in animals and humans (2).

Cases of *V. gallopava* infection have been reported in immunocompromised patients, particularly solid organ transplant (SOT) recipients, individuals with hematologic malignancies, HIV infection, and chronic granulomatous disease (3, 4). The infection typically manifests as pulmonary or disseminated disease, often involving the central nervous system. High-risk individuals, including those with significant environmental exposure such as gardeners and woodworkers, may acquire *V. gallopava* through inhalation of fungal spores or, less commonly, through direct inoculation through wounds (5).

Isavuconazole (ISA) is a relatively new triazole with broad spectrum activity against invasive mold species. However, clinical breakthrough infections have been increasingly recognized in recent literature (6). While ISA has a favorable adverse event profile, its clinical utility remains limited in high-risk patients despite demonstrating good *in vitro* efficacy.

Treatment guidance for rare dematiaceous molds such as *V. gallopava* remains sparse, with limited data comparing the efficacy of ISA to other mold-active azoles, such as voriconazole or posaconazole (POS). Moreover, minimum inhibitory concentrations (MICs) and standardized breakpoints for ISA against dematiaceous fungi have not been established. Given these challenges, we present a case to contribute to the growing clinical experience in diagnosing and managing infections caused by *V. gallopava*.

## CASE PRESENTATION

A 52-year-old male presented with a two-week history of progressively worsening productive cough of green-tan sputum. His symptoms were accompanied by right-sided pleuritic chest pain, dyspnea, fever, chills, and profound fatigue.

His past medical history was significant for coronary artery disease with ischemic cardiomyopathy, implantable cardioverter-defibrillator, severe mitral regurgitation status post mechanical mitral valve replacement, obstructive sleep apnea, and chronic kidney disease.

Ten weeks before his presentation, he had undergone orthotopic heart transplantation. His immediate post-transplant course was complicated by right ventricular dysfunction requiring inotropic support and continuous renal replacement therapy. His induction regimen included basiliximab, and maintenance immunosuppression consisted of tacrolimus, prednisone, and mycophenolate mofetil.

Post-transplant antimicrobial prophylaxis was initiated based on his infectious risk profile. He received valganciclovir for cytomegalovirus prophylaxis and atovaquone for *Pneumocystis jirovecii* pneumonia prophylaxis. As per institutional protocol for patients requiring renal replacement therapy postoperatively, fungal prophylaxis with ISA is recommended for 3 months, with voriconazole as an alternative option. The patient no longer required renal replacement therapy and was not considered at high risk for disseminated fungal infection. Consequently, antifungal management was de-escalated, and the patient was transitioned to nystatin swish and swallow to continue for 3 months.

On presentation, he was afebrile but tachycardic, tachypneic, and hypoxic, requiring supplemental oxygen via nasal cannula with paroxysmal coughing spells and diffuse rhonchi with diminished air entry throughout all lung fields on exam. No rashes were noted and did not have any mental status changes.

**Fig 1:**
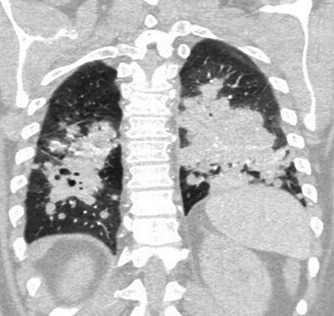
Coronal CT angiogram of the chest on admission. Widespread nodular consolidation through both lung fields, which has a cavitary component in the right lower lobe.

Biochemical analyses are presented in Table 1. Chest X-ray demonstrated bilateral perihilar consolidations, more pronounced on the right. Computed tomography (CT) of the chest revealed extensive bilateral nodular consolidation with a cavitary lesion in the right lower lobe (Fig. 1).

**TABLE 1:** Laboratory data from admission

Blood		
White blood cell count	7.09 k/μL	3.60–11.0 k/μL
Hemoglobin	9.2 g/L	14.0–18.0 g/L
Platelets	559 k/μL	130–400 k/μL
Sodium	134 mmol/L	136–144 mmol/L
Chloride	98 mmol/L	98–109 mmol/L
Glucose	266 mg/dL	74–106mg/dL
Creatinine	1.5 mg/dL	0.6–1.3mg/dL
Blood urea nitrogen	19 mg/dL	8–26 mg/dL
Brain natriuretic peptide	224 pg/mL	0–100 pg/mL
Troponin	10 ng/L	<27 ng/L
Serum 1,3 beta-D-glucan	>500 pg/mL	<60 pg/mL
Serum cryptococcus antigen	Negative	Negative
*Aspergillus* galactomannan antigen	Negative	Negative

Urine		
Urine *Histoplasma* antigen	Negative	Negative
Urine *Blastomyces* antigen	Negative	Negative

His social history was not significant for any animal exposure or pets in the home. He worked as a short-haul truck driver, a longtime carpenter, and recently had been coaching football.

A review of the donor’s medical history, screening tests, cultures, and imaging revealed no risk factors for donor-derived fungal infection, with no evidence of fungal colonization or infection in the donor’s lungs or bloodstream.

Blood cultures remained negative. Five expectorated sputum samples were collected over his first three hospital days and sent for bacteria, fungal, and acid-fast smears and cultures. Bronchoscopy with bronchoalveolar lavage (BAL) sampling was performed for the same microbiological test in addition to cytology.

**Fig 2:**
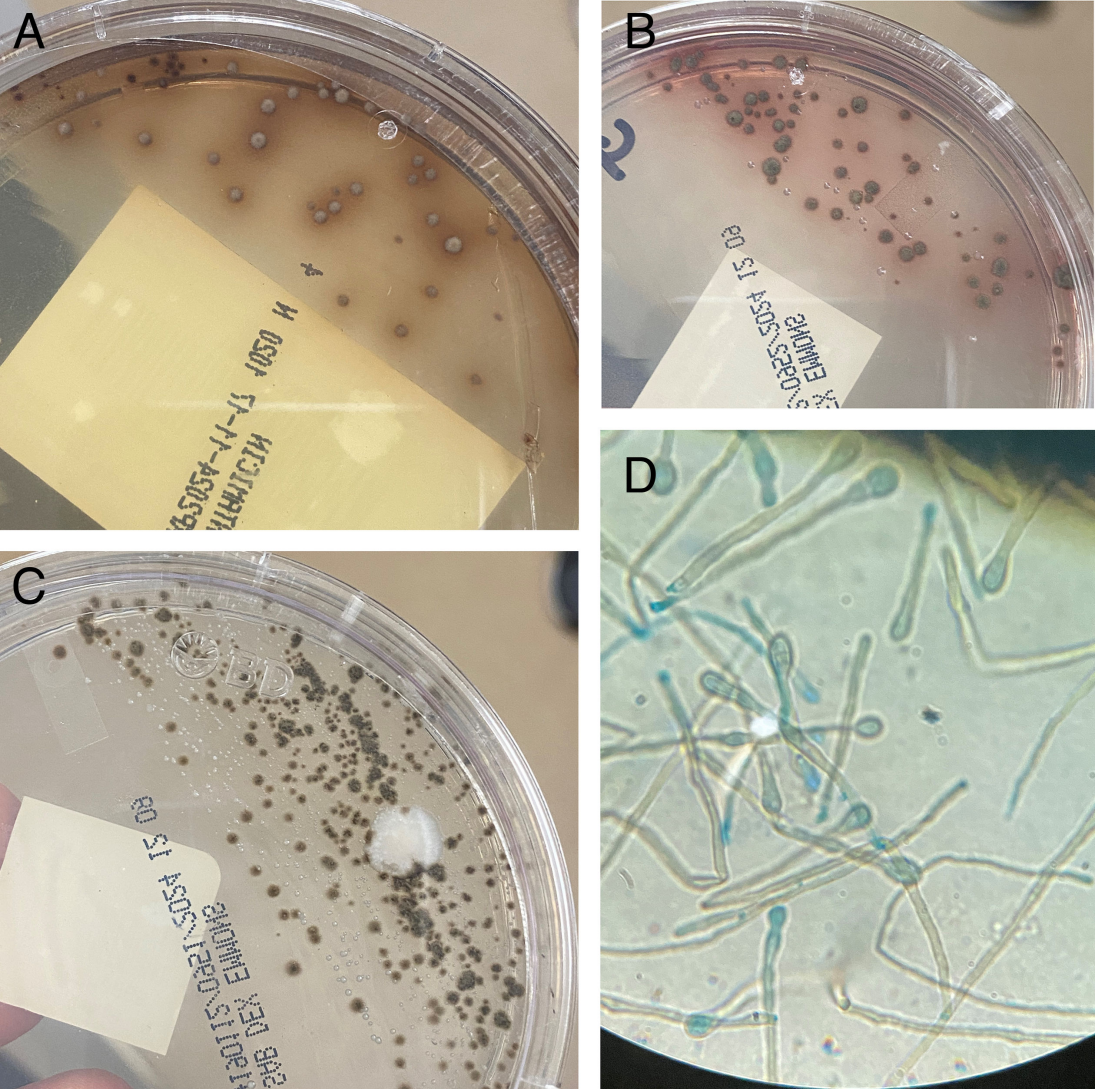
Appearance of smooth, brown to darkly pigmented mold colonies at 3 days of incubation at 35°C with diffusion of pigment into the agar on inhibitory mold agar with gentamicin (**A**) and Sabouraud’s dextrose agar (**B**) from the patient’s BAL specimen from hospital day 2. Pigment is visible on both the obverse (shown) and reverse (not shown) of the cultures. A similar appearance (heavy growth mixed with *Candida* spp. and a single colony of *Mycobacterium abscessus* ssp. *abscessus*) is noted in the fungal cultures of expectorated sputum from his first hospital day (**C**). Lactophenol cotton blue staining (**D**) of a tape preparation from 3-day-old colonies shows brown-pigmented hyphae with two-celled conidia, with the apical cell wider than the basal cell is most seen.

An empiric regimen of meropenem and ISA was initiated after the patient, who initially presented to an outside hospital, failed to defervesce while receiving cefepime and vancomycin. The rationale for the initiation of ISA at the outside hospital is unclear; however, the therapy was continued upon arrival due to the patient’s prior tolerability. However, the patient showed no clinical improvement after 6 days of treatment. On hospital day 7, sputum fungal cultures revealed the growth of a darkly pigmented, melanin-producing (dematiaceous) mold with morphology suggestive of *Verruconis* species (Fig. 2) on both BAL and sputum fungal culture specimens. Therefore, meropenem and ISA were discontinued, and the antifungal regimen was switched to daily, 5 mg/kg IV liposomal amphotericin B.

After 3 days of treatment, the patient showed mild symptomatic improvement. For discharge planning, ISA was reintroduced, as it could be administered orally and did not require close monitoring. However, 6 days after the transition, his condition worsened, with recurrent fever, worsening shortness of breath, and progressive hypoxia. A repeat CT chest revealed increasing nodular consolidations. At this time, the identification of the mold species and antimicrobial susceptibility data remained pending.

Given the patient’s clinical deterioration and the suspected dematiaceous mold infection, an MRI of the brain was obtained to assess central nervous system involvement. While no evidence of CNS disease was identified, his respiratory status continued to decline despite therapeutic serum trough target levels of ISA (5.8 mcg/mL). After 7 days of ISA monotherapy, intravenous liposomal amphotericin B was reintroduced, resulting in gradual clinical improvement, with slow defervescence of fever and resolution of cough and dyspnea. Due to concerns regarding antifungal resistance, ISA was switched to POS, and repeat CT demonstrated stable disease.

Fungal identification and susceptibilities were performed at South Texas Reference Laboratory through the University of Texas Health Center at San Antonio. The isolate was identified as *V. gallopava* through a combination of phenotypic characterization and DNA sequencing, specifically targeting the ITS and D1/D2 regions. Antifungal susceptibility testing revealed an ISA MIC of 4 mcg/mL, whereas POS demonstrated an MIC of <0.03 mcg/mL (Table 2). The patient completed a 10-day induction course of liposomal amphotericin B, after which he was discharged on POS monotherapy with therapeutic drug target of >1200 ng/mL.

**TABLE 2:** Antifungal susceptibility results (method CLSI M38)^*a*^

Drug	Result (mcg/mL)
Amphotericin B (AMB)	≤0.03
Micafungin (MICA)	0.03
Fluconazole (FLU)	>64
Itraconazole (ITRA)	≤0.03
Posaconazole (POS)	≤0.03
Voriconazole (VORI)	0.5
Isavuconazole (ISA)	4
Terbinafine (TERB)	0.015

^*a*^ Breakpoints and interpretation are not established for this species.

**Fig 3:**
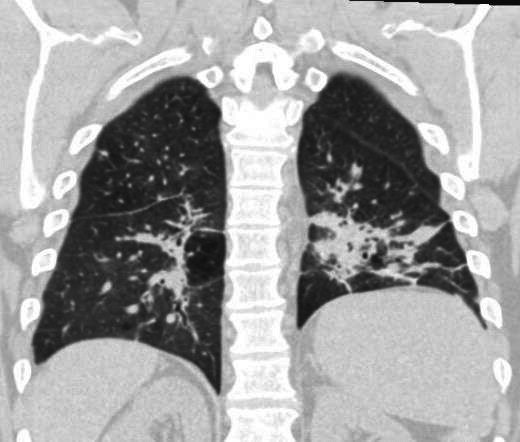
Coronal CT chest with significant improving consolidations and nodular infiltrates in both lungs.

The patient tolerated 4 months of POS therapy with improvement in both respiratory symptoms and nodular opacities on CT (Fig. 3).

## DISCUSSION

The patient presented as a rare case of phaeohyphomycosis in an immunocompromised host. Patient risk factors included work as a carpenter with exposure to sawdust and outdoor activity as a football coach. He presented within 10 weeks of undergoing heart transplantation with a symptomatic cough. This timeline is significantly shorter than the median onset of *V. gallopava* infection reported in SOT recipients, which was previously reported as 18 months (7). The relatively early onset indicates the infection was likely acquired before transplantation, with post-transplant immunosuppression enabling its reactivation and progression to clinical disease. Alternatively, it may reflect exposure to a high pathogen inoculum in an immunocompromised host, leading to rapid disease development.

This case highlights the potential limitations of ISA as an empiric therapy for an invasive fungal infection treatment and prophylaxis in SOT, as no standardized treatment guidelines exist regarding the choice of antifungal therapy or the optimal duration of treatment for *V. gallopava* infections. High MICs to ISA have been reported in the literature, thus suggesting an alternative mold-active triazole should be considered for empirical therapy (8). In this case, management was guided by a previously reported case of pulmonary phaeohyphomycosis in a heart transplant recipient, successfully treated with POS monotherapy (2); however, antifungal selection in our patient remained empiric until definitive mold identification and susceptibility data became available.
